# The Effect of Copper Adsorption on Iron Oxide Magnetic Nanoparticles Embedded in a Sodium Alginate Bead

**DOI:** 10.3390/nano15151196

**Published:** 2025-08-05

**Authors:** Michele Modestino, Armando Galluzzi, Marco Barozzi, Sabrina Copelli, Francesco Daniele, Eleonora Russo, Elisabetta Sieni, Paolo Sgarbossa, Patrizia Lamberti, Massimiliano Polichetti

**Affiliations:** 1 Department of Physics, University of Salerno, Via Giovanni Paolo II 132, 84084 Fisciano, Italy; mmodestino@unisa.it (M.M.); agalluzzi@unisa.it (A.G.); 2 Science and High Technology Department, Insubria University, Via Valleggio 11, 22100 Como, Italy; marco.barozzi@uninsubria.it (M.B.); sabrina.copelli@uninsubria.it (S.C.); 3 Theoretical and Applied Science Department, Insubria University, Via O. Rossi 9, 21100 Varese, Italy; fdaniele@uninsubria.it; 4 Industrial Engineering Department, Padova University, Via F. Marzolo 9, 35122 Padova, Italy; eleonora.russo.2@phd.unipd.it (E.R.); paolo.sgarbossa@unipd.it (P.S.); 5 Department of Information and Electrical Engineering and Applied Mathematics, University of Salerno, 84084 Fisciano, Italy; plamberti@unisa.it

**Keywords:** copper adsorption, sodium alginate beads, magnetic properties, magnetic nanomaterials

## Abstract

The preparation and use of iron oxide magnetic nanoparticles for water remediation is a widely investigated research field. To improve the efficacy of such nanomaterials, different synthetic processes and functionalization methods have been developed in the framework of green chemistry to exploit their magnetic properties and adsorption capacity in a sustainable way. In this work, iron oxide magnetic nanoparticles embedded in cross-linked sodium alginate beads designed to clean water from metal ions were magnetically characterized. In particular, the effect of copper adsorption on their magnetic properties was investigated. The magnetic characterization in a DC field of the beads before adsorption showed the presence of a superparamagnetic state at 300 K—a state that was also preserved after copper adsorption. The main differences in terms of magnetic properties before and after Cu^2+^ adsorption were the reduction of the magnetic signal (observed by comparing the saturation magnetization) and a different shape of the blocking temperature distribution obtained by magnetization versus temperature measurements. The evaluation of the reduction in magnetization can be important from the application perspective since it can affect the efficiency of the beads’ removal from the water medium after treatment.

## 1. Introduction

The worldwide diffusion of industrialization has led to an increase in the presence of metals and metalloids in water. This is due to their use in processes like electrolysis, electroplating, and metal smelting, resulting in a contamination of the environment whenever the wastewater treatment methods are insufficient for their removal [1]. The effects of metals and metalloids, such as copper (Cu), iron (Fe), zinc (Zn), nickel (Ni), and arsenic (As), on human health are well known, and they should not be underestimated [2,3,4]. In the attempt to reduce as much as possible their concentration, if not to completely remove them from water bodies, many different technologies have been proposed and developed. Methods such as ion exchange, membrane filtration, and chemical precipitation are widely used, but their applications have disadvantages in terms of either economic and working costs or use of secondary chemicals [1,5]. Thus, new and sustainable methods based on non-environmentally impacting materials are necessary. In this framework, magnetic nanoparticles have been of great interest and a research focus due to a combination of unique magnetic properties and the possibility of functionalization [1,6,7,8]. Magnetic nanoparticles are ferromagnetic or antiferromagnetic materials that, as a result of their small dimension (with a diameter below c.a. 50 nm, depending on the material), can show superparamagnetic properties at room temperature. These properties are characterized by an absence of coercivity, as expected for a paramagnet, but with a magnetic signal with intensity comparable to ferromagnets [9,10,11]. Superparamagnetic properties are used in most applications concerning magnetic nanoparticles, from biomedical ones to wastewater remediation [12,13,14,15,16]. For these applications, the possibility of functionalizing the magnetic nanoparticles with an organic coating or embedding them in a polymer matrix is fundamental, giving rise to the possibility of exploiting the properties of the organic component, such as the biocompatibility of metal ions complexation. In fact, the use of magnetic nanoparticles for wastewater remediation has been extensively considered thanks to the possibility of choosing proper coatings for metal and metalloid adsorption [8]. The advantages of this application are the simplicity and high efficiency of the adsorption process, as well as the possibility of easily removing the nanoadsorbents after purification to regenerate and reutilize them [5,7,17]. With respect to the incorporation in polymers, this opens the possibility of preparing a wealth of hybrid materials, with the nanoparticles imparting useful magnetic properties. One example is the incorporation of magnetic nanoparticles in alginates [18,19]. The polysaccharide of natural origin and its modified or functionalized derivatives can easily form cross-linked hydrogels with good mechanical properties that, as small beads, can be used in wastewater remediation for their high adsorption capacity [20]. The advantages connected to the use of these materials are, again, low cost and the possibility of recovery and regeneration, particularly if they are integrated with magnetic nanoparticles, enabling the easy separation from water. In this work, sodium alginate beads with two types of incorporated iron oxide magnetic nanomaterials (namely, magnetite nanoparticles, MNPs, and graphene oxide decorated with MNPs) were prepared. Those with only MNPs were magnetically characterized and compared, before and after copper(II) ions adsorption, with the pristine magnetic nanoparticles. The intent is to evaluate how the adsorption of copper ions influences the magnetic properties of the embedded nanoparticles.

## 2. Materials and Methods

All reagents and solvents were purchased from Sigma-Aldrich, St. Louis, MO, USA, unless otherwise indicated, and used without further purification. Magnetite nanoparticles (MNPs) were prepared according to the protocols described in the previous publications of the same authors [21,22] and magnetically analyzed as a reference.

### 2.1. Alginate Beads Preparation

**Figure 1:**
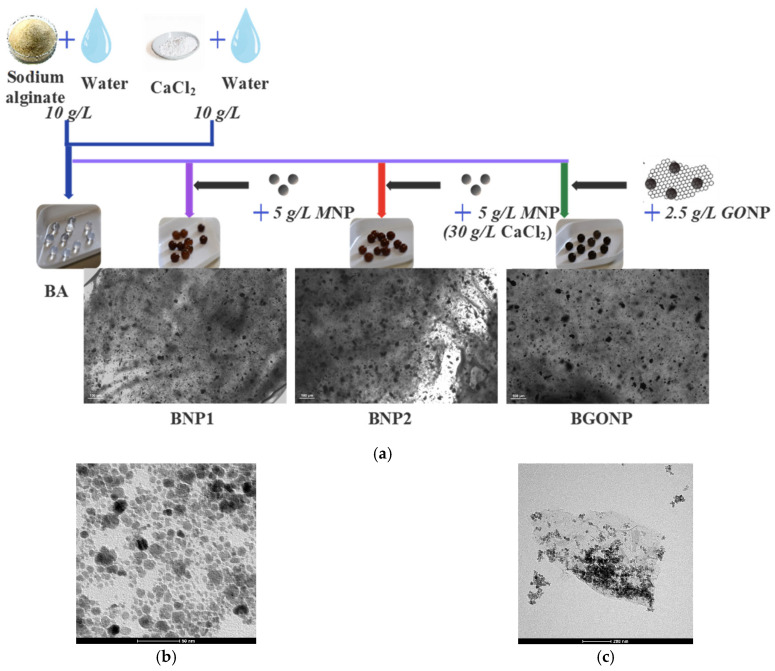
(**a**) The preparation scheme of the adsorbent beads. From left to right: BA (pure alginate beads), BNP1 (alginate beads with embedded MNPs), BNP2 (alginate beads with embedded MNPs and a higher concentration of hardener), BGONP (alginate beads with embedded GONPs), along with a 40× picture of their section at a confocal microscope (with white bar scale 100 μm long). TEM images of the magnetic nanomaterials used in the preparation of the beads (**b**) BNP1 (the NPs in BNP2 were similar and are not shown), and (**c**) BGONP with detail of graphene oxide sheet, respectively (scale impressed in the figure).

The preparation of nanoadsorbents is outlined in Figure 1: the iron oxide MNPs and the graphene oxide decorated with MNPs (GONP) were prepared by coprecipitation from an alkaline solution, as reported elsewhere [21,22], and included in alginate beads, as outlined below. The composite adsorbents were obtained in the form of spherical hydrogel beads with a diameter around 4 mm. Figure 1 shows the confocal microscope (Olympus IX51, manufactured by Olympus Scientific Solutions, part of Evident Corporation, Tokyo, Japan) images (Figure 1a) and TEM (Transmission Electron Microscope, model TECNAI FEI G2, FEI company, Hillsboro, OR, USA) images (Figure 1b,c) of the nanoparticles in the beads with a detail of the graphene oxide sheet decorated with nanoparticles (Figure 1c).

Depending on both the presence and type of magnetic nanomaterial filler, four types of beads were prepared and tested:

- Pure alginate beads (sample BA);
- Alginate beads with MNPs at different hardener (calcium chloride) solution concentrations (samples BNP1 and BNP2);
- Alginate beads with GONP (named BGONP).

Samples BNP1 and BNP2 were prepared following the method described in [21] and herein briefly resumed:

(1) Preparation of the sodium alginate solutions (10.0 g/L) with suspended MNPs (5.0 g/L for BNP1 and BNP2, 2.5 g/L for BGONP) by ultrasonication.
(2) Drop-wise addition of each suspension in a cross-linking solution, obtained by dissolving either 10.0 g/L (for BA, BNP1, and BGONP) or 30.0 g/L (for BNP2) of calcium chloride (CaCl_2_) in deionized water. During the addition, the Ca^2+^ solution was kept under constant agitation.
(3) Collection of the formed hydrated beads from the solution, followed by washing four times with deionized water, and storage in deionized water prior to use.

In particular, in BNP1 and BNP2, the same type of magnetic nanoparticles was embedded (the TEM in Figure 1b shows the NPs embedded in BNP1, and Figure 1c shows the GO sheet decorated with nanoparticle embedded in BGONP).

### 2.2. Experimental Procedure for Sample Preparation

According to the authors’ previous work [21,22], different beads were tested on water samples containing Cu^2+^ ions obtained by diluting copper(II) sulphate pentahydrate (CuSO_4_∙5H_2_O, 99%) in milli-Q water. The beads loaded with copper have been prepared by adding 10 beads in 4 mL glass vials, where 2 mL of a solution at a defined copper concentration was added. The beads have been mixed for 24 h, and then, the solution was analyzed to establish the final Cu^2+^ concentration. The copper content on the beads was found by considering the difference between the initial and final copper found in the samples.

### 2.3. Spectrophotometric Determination of Metal Load

Metal ion loads were estimated by measuring the concentration of copper(II) in solution via absorbance by UV-VIS spectrophotometry (Jasco V-560 Spectrophotometer, Jaco Europe s.r.l., Cremella, Italy) with optical glass cuvettes. The use of such an analytical technique was previously validated by comparing the measured ion concentrations with other widely used techniques, such as ICP (Inductively Coupled Plasma). For Cu^2+^, results showed a deviation below 4%, therefore justifying the use of such a technique for the aim of the present work.

**Figure 2:**
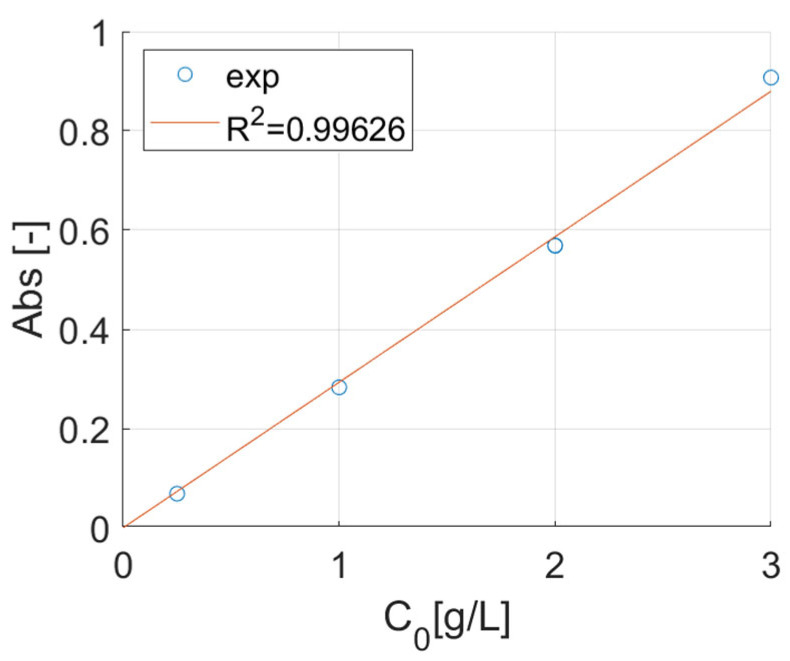
The calibration curve for copper (II) stock solutions.

The absorbance was measured at the maximum peak corresponding to 780 nm [23]. A calibration curve was created using a linear regression through the Beer–Lambert law by measuring a series of Cu^2+^ stock solutions at 3.00–2.00–1.00–0.25 g/L (each measurement was repeated 3 times, and the average value was taken). The stock solutions were prepared by dissolving copper(II) sulphate pentahydrate in milli-Q water. The pH of the solution was not corrected and was measured at values between 4.5 and 5 in all the solutions. Figure 2 shows the calibration curve with the determination coefficient (R^2^).

The load tests were conducted according to the following protocol: a series of 4 mL vials were loaded with 2 mL of Cu^2+^ stock solution (0.25–1.00–2.00–3.00 g/L), 10 beads (of a single type per vial) and left for 24 h at 25 °C to ensure the equilibrium was reached. After the test, the treated water was analyzed by spectrophotometry to determine the metal concentration, and the copper(II) load was calculated according to the following equation:

$$x_{load}\left[\frac{mg_{metal}}{g_{bead}}\right]=\frac{C_{0}\cdot C_{eq}}{n\cdot m_{bead}}\cdot V\cdot 1000$$

where $x_{load}$ [mg/g] is the amount of metal adsorbed per gram of bead. $C_{eq}$ [g/L] is the equilibrium concentration of the metal in solution after the treatment, $C_{0}$ [g/L] is the metal starting concentration, n is the number of beads per vial, $m_{bead}$ [g] is the average mass of each bead (determined from Table 1), V [L] is the volume of solution in the vial.

After treatment, the beads were isolated and dried prior to undergoing the magnetic measurements.

### 2.4. Magnetism Analysis

The magnetic characterization of the samples was performed by means of a Physical Properties Measurement System (PPMS) by Quantum Design equipped with a Vibrating Sample Magnetometer (VSM). The PPMS has a superconductor magnet that reaches a DC magnetic field of 90,000 Oe and can perform measurements in a temperature range from 1.9 K to 400 K. The samples were characterized by studying the magnetization as a function of both the applied DC field, M(H), and temperature, M(T). The M(H)s were measured at 300 K and 5 K by varying the applied field from 0 Oe to 90,000 Oe, then by looping it from 90,000 Oe to −90,000 Oe and back to 90,000 Oe. The M(T)s were measured with an applied DC field of 100 Oe and by varying temperature from 5 K to 300 K. Before both measurements, the trapped field in the superconductor magnet was reduced by following the procedure described in reference [24]. The M(T)s were measured with both Zero Field Cooling (ZFC) and Field Cooling (FC) protocols. For the ZFC protocol, the temperature was reduced after field zeroing, then a magnetic field of 100 Oe was applied, and the magnetization was measured by increasing the temperature to 300 K. Once 300 K was reached, to follow the FC protocol, the magnetization was acquired by reducing the temperature to 5 K without turning off the applied field.

## 3. Results

By applying an easy and reproducible preparation procedure, three types of nanocomposite adsorbent beads based on cross-linked alginate loaded with magnetite nanoparticles or MNP-decorated graphene oxide were prepared. They were compared with pure cross-linked alginate beads in the adsorption of copper(II) ions in aqueous solution to assess the effect of metal adsorption on the magnetic properties of the material.

In order to properly assess the equilibrium load of all the beads, the net mass of adsorbing material had to be determined. Since the produced alginate beads were in the form of a hydrogel, composed of up to 95% water, a sample of 20 to 40 beads per type was taken and lyophilized for 24 h. Table 1 reports the mass of wet vs. dry beads. The average dried mass was used to evaluate the load of copper(II) per gram of bead.

### 3.1. Isotherms Characterization

Batch isotherms were developed according to the following protocol: 5 beads were placed in a vial with 2 mL of copper(II) sulphate solution at 0.25, 1.00, 2.00, and 3.00 g/L (with respect to copper), and stirred for 1 h in a platform rocker at controlled temperature (25, 35, 45 °C). After adsorption was finished, the final solution was collected at once and sent to the spectrophotometer for analysis. From the data, isotherms according to the Langmuir model were developed. Both unbuffered solutions (pH between 4.5 and 5, due to copper(II) being a conjugated acid of a weak base, 3 repetitions for each concentration), and pH 3 (corrected with HCl, 2 repetitions for each concentration) were analyzed. Temperature dependence was studied at pH equal to 3. Table 2 reports the results of the maximum load Q_max_ (expressed as mg_Cu_/g_bead_) according to the Langmuir adsorption model.

**Figure 3:**
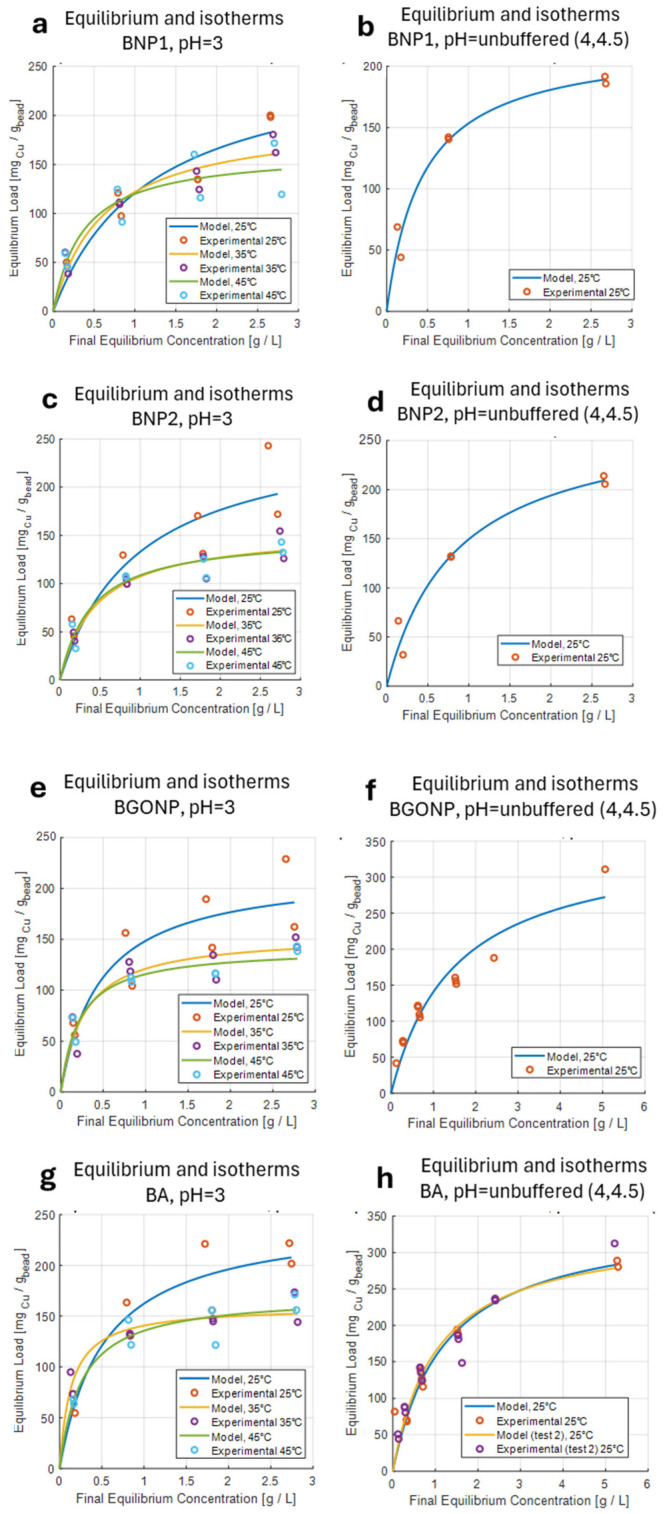
Adsorption equilibrium isotherms for copper under different conditions. (**a**) BNP1, pH 3; (**b**) BNP1, unbuffered pH; (**c**) BNP2, pH 3; (**d**) BNP2, unbuffered pH; (**e**) BGONP, pH 3; (**f**) BGONP, unbuffered pH; (**g**) BA, pH 3; (**h**) BA, unbuffered pH.

Figure 3 reports both the experimental results and the fitting of the Langmuir isotherms. According to the achieved results, for each bead analyzed in buffered conditions at pH 3, higher temperatures show a general decrease in the maximum load, a behavior consistent with most chemical and physical adsorption isotherm models. This is also observed in other adsorbents bearing protonated carboxylic acid groups [25] in which the release of the exchangeable protons caused the desorption of Cu(II). The importance of the protonation–deprotonation equilibrium is also proved by observing the effect of pH, which generally shows an increase in the maximum load with unbuffered solutions. In fact, at acidic pH, the carboxylic groups are partially protonated, limiting their coordination ability/ionic interaction toward the copper cations. BGONP and BA showed the maximum increase in load, so we added adsorption tests at 5 g/L of copper(II) to ensure a proper estimation of the maximum load under equilibrium conditions. This is due to the fact that graphene oxide can contribute to the adsorption of copper ions thanks to the oxygenated functional groups in its structure.

### 3.2. Copper(II) Load on Beads

The equilibrium load of copper(II) on the four types of adsorbent beads was measured by spectrophotometric determination of the metal concentration in solution after 24 h of contact time with a selected number of beads. The results at different Cu^2+^ starting concentrations are reported in Table 3 in terms of the concentration of metal ions adsorbed per gram of beads. It can be deduced from Table 3 that the load determined on the beads is compatible with the maximum load found with the isotherms developed.

Copper(II) exhibited a comparable affinity with BNP1 and BNP2, as previously observed [21], resulting in similar loads, lower than pure alginate (BA), probably due to the reduced amount of alginate and, thus, of exchangeable divalent cations. However, BGONP displayed a similar load to BA thanks to the contribution of graphene oxide as an adsorbent co-filler. The loaded bead of type BNP1 was extracted from the solution with 3.00 g/L of Cu^2+^ concentration after treatment, washed with milli-q water, and dried for the magnetic characterization.

### 3.3. Magnetic Characterization

**Figure 4:**
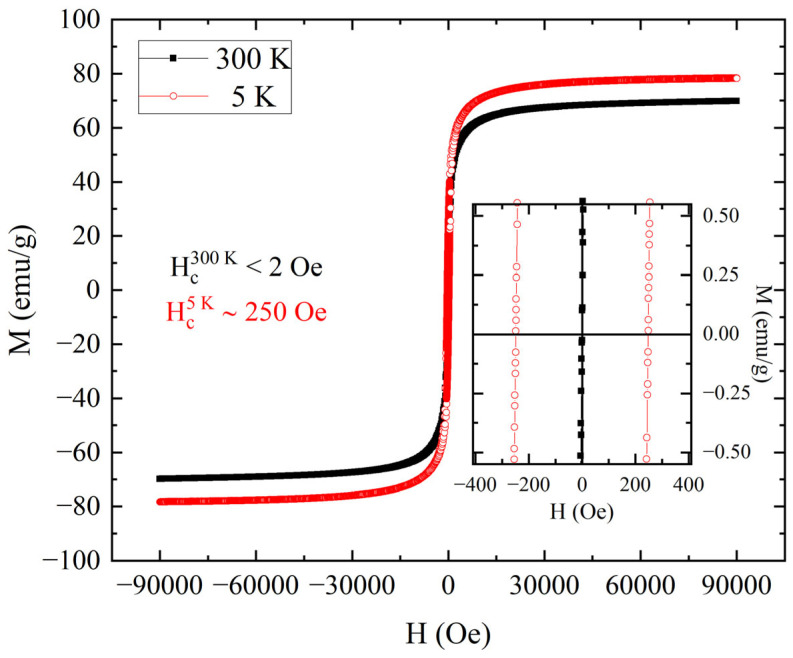
M(H) loops performed at 5 K (red open circles) and 300 K (black closed squares) for the powder of Fe_3_O_4_ magnetic nanoparticles. The inset shows the data near to zero field. From this, it is possible to evaluate the coercive field at 300 K as low as 2 Oe and the coercive field at 5 K of about 250 Oe.

The curves obtained by performing the M(H) measurements on the powder of Fe_3_O_4_ magnetic nanoparticles at 5 K and 300 K are reported in Figure 4. The value of the mass magnetization at 90,000 Oe (M_s_) was about 70 emu/g at 300 K and about 78 emu/g at 5 K. These values are comparable to the ones reported in the literature for samples of Fe_3_O_4_ nanoparticles [14,19,26]. The increase in mass magnetization at 5 K and the change in coercivity field from a value below 2 Oe at 300 K to a value about 250 Oe at 5 K suggested the presence of a superparamagnetic behavior at the highest temperatures [11]. In fact, despite the absence of a coercive field, which is expected for a paramagnet, the shape of the loop at 300 K showed a rapid increase in magnetization below a field intensity of 10,000 Oe, as expected for the signal of a ferromagnet.

**Figure 5:**
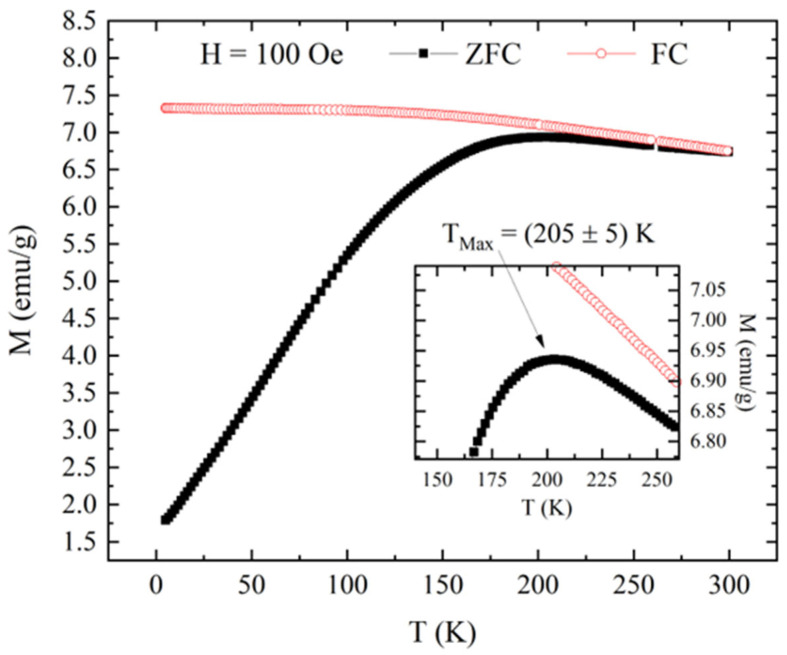
Magnetization versus temperature measured with an applied field of 100 Oe in the ZFC (black closed squares) and FC protocol (red open circles) for a powder of Fe_3_O_4_ magnetic nanoparticles. The inset shows the behavior around the peak of the ZFC curve, showing a maximum associated temperature of 205 K.

To better investigate the transition from a blocked state to a superparamagnetic behavior for the powder of Fe_3_O_4_ magnetic nanoparticles, the study of magnetization versus temperature with 100 Oe of applied field was performed, and the curves obtained are reported in Figure 5. The inset reveals that the ZFC curve reaches its maximum (T_Max_) at a temperature of 205 K. This T_Max_ represents the mean blocking temperature for the sample of magnetic nanoparticles, indicating a transition to a superparamagnetic behavior [27,28]. In fact, as observed in Figure 5, at temperatures below T_Max,_ the ZFC and FC curves were completely separated, as expected for a sample of magnetic nanoparticles in a blocked state. For temperatures above T_Max_, the separation between ZFC and FC curves suggested that not all the nanoparticles were in a superparamagnetic state [28]. The two curves had an overlap at near room temperature, indicating that the sample had a reversible behavior. This was an indication of the complete transition to the superparamagnetic state, and it is in accordance with the absence of hysteretic behavior at 300 K (see inset of Figure 4).

**Figure 6:**
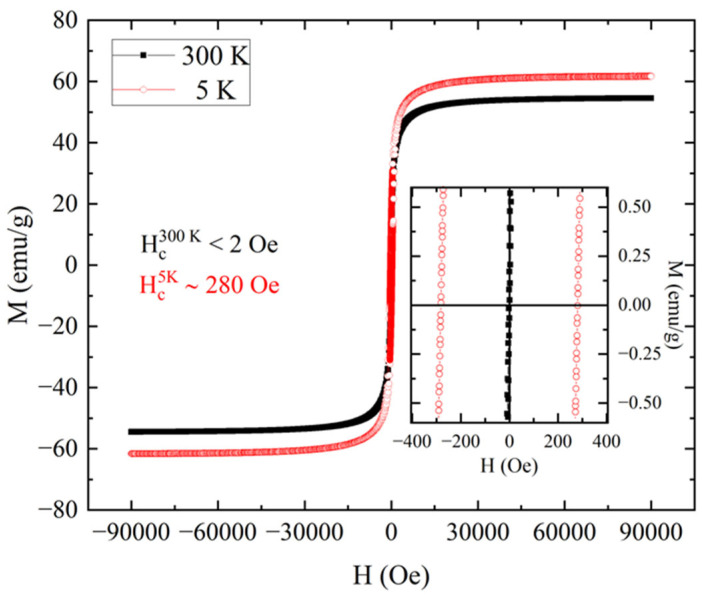
M(H) loops performed at 5 K (red open circles) and 300 K (black closed squares) for the BNP1 bead. The inset shows the data near to zero field. From this, it is possible to evaluate the coercive field at 300 K as 2 Oe and the coercive field at 5 K of about 280 Oe.

The same magnetic characterizations were performed on a bead BNP1 containing the same magnetic nanoparticles shown in Figure 4 and Figure 5. To compare the magnetic properties of the pristine nanoparticles and their alginate composite beads, the sample BNP1 was chosen. This would allow for highlighting the effect of the embedment in the polysaccharide matrix. The M(H) loops obtained for the dried bead at 300 K and 5 K are reported in Figure 6. The mass magnetization was obtained by normalizing the magnetic moment with the mass of magnetic nanoparticles embedded in the sodium alginate. Considering the mass proportion between magnetic nanoparticles and sodium alginate determined for BNP1 in [21], the mass of the former was estimated to be one-third of the total dry mass of the bead. The first observation that can be made from Figure 6 is that the saturation magnetization at 300 K was lower than that at 5 K. Furthermore, from the inset, which showed the data around zero field, it was possible to evaluate the coercivity of the sample. In particular, the coercive field at 5 K was about 280 Oe, while the coercive field at 300 K was lower than 2 Oe. This reduction in coercivity suggests that magnetic nanoparticles retained their superparamagnetic behavior also when they were embedded in the bead. Despite this, there was a difference in the mass magnetization at 90,000 Oe, both at 5 K and 300 K for the two samples. From Figure 6, the mass magnetization reached a maximum of 54 emu/g at 300 K and of 62 emu/g at 5 K. Comparing these values with those measured in Figure 4 for the powder of magnetic nanoparticles (70 emu/g at 300 K and 78 emu/g at 5 K), it was possible to see a difference of around 16 emu/g at each temperature (reduction of 23% at 300 K and of 21% at 5 K). The mass ratio of 1/3 between magnetic nanoparticles and the total mass of the bead, used to evaluate the mass magnetization, was obtained considering the proportion between magnetic nanoparticles and sodium alginate in the synthesis process of the bead. For this reason, the one-third fraction is an average value but could slightly change when considering a single bead. This could explain the difference between the M_s_ of the pure powder of magnetic nanoparticles and the bead. Another possible explanation could be the presence of an alternating surface anisotropy due to the incorporation of nanoparticles in the sodium alginate matrix. Despite this, the value obtained for M_s_ was comparable to that reported in the literature for similar types of beads [19] produced for wastewater remediation.

**Figure 7:**
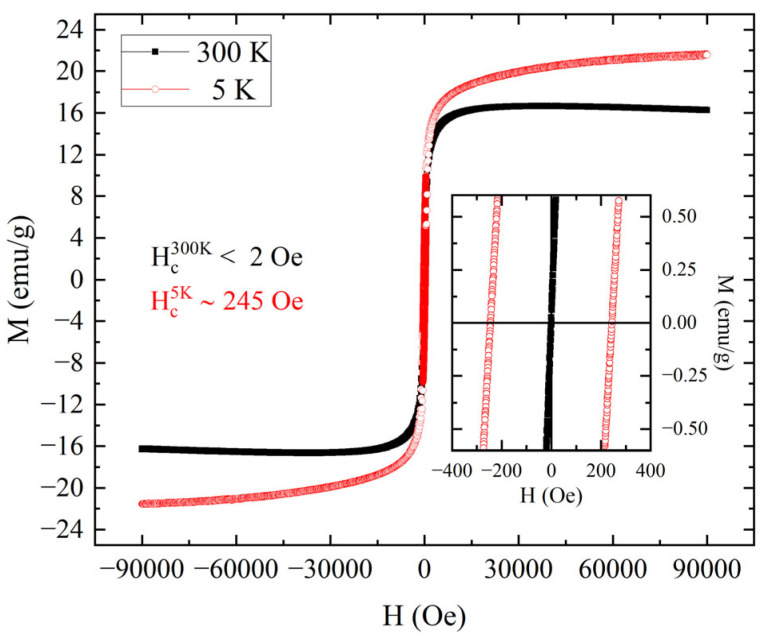
Magnetization versus field loops performed at 5 K (red open circles) and 300 K (black closed squares) for the BNP1 bead after copper ions adsorption. The inset shows the data near to zero field. From this, it is possible to evaluate the coercive field at 300 K as low as 2 Oe and the coercive field at 5 K of about 245 Oe.

Focusing on this latter application, the magnetic properties of a bead of BNP1type dried after adsorbing copper ions (in a 3.00 g/L Cu^2+^ solution) were analyzed by performing the M(H) measurements (Figure 7). To maximize the metal effect on the adsorbent’s magnetic signal, the BNP1 sample with the highest copper load was selected for this characterization. From the M(H) shown in Figure 7, it was possible to observe that the superparamagnetic behavior at 300 K remained after the adsorption of copper ions. In fact, the coercive field changed from about 245 Oe at 5 K to a value below 2 Oe at 300 K. This difference was perfectly in accordance with the behavior observed in Figure 4 and Figure 6. Despite this, the magnetic responses showed a difference in terms of signal intensity. In fact, as it can be seen in Figure 7, the maximum of mass magnetization for the bead after adsorption was 16 emu/g at 300 K and 21 emu/g at 5 K, with a difference of more than 40 emu/g with the data reported in Figure 6 (a reduction of 74% at 300 K and 65% at 5 K). One possibility to explain the change in the signal is to consider that the value of mass used to calculate mass magnetization in Figure 7 was higher than that used for Figure 6. In fact, the mass magnetization in Figure 7 was calculated by considering one-third of the mass of the dried bead after copper adsorption, exactly as in the case of a dried bead before adsorption; however, in this case (after the adsorption), the total mass of the bead considered the presence of not only sodium alginate and magnetic nanoparticles but also copper ions. So, there was an error in the estimation of the mass associated with magnetic nanoparticles due to the presence of copper. In fact, the average mass of a group of 20 beads before copper adsorption and of 20 beads after copper adsorption differed by around 0.5 mg. However, even rescaling the mass used for normalization in Figure 7 to the one used for the bead before adsorption (Figure 6), M_s_ would be 21 emu/g at 300 K and 27 emu/g at 300 K. Thus, the difference in the case of bead before adsorption would be more than 30 emu/g (a reduction of 55% at 300 K and 47% at 5 K). Another possibility to explain this change in the signal could be to consider the signal associated with the presence of copper inside the bead. Considering that the mass susceptibility of bulk copper at 300 K was −0.0830 × 10^−6^ emu/(Oe g) [29] and evaluating the signal associated with the estimated mass of 0.5 mg of copper at 300 K with an applied field of 90,000 Oe, the contribution of copper would be about −4 × 10^−6^ emu and about −0.017 emu/g after the normalization with mass used for data in Figure 7. From this estimation, a reduction of about 0.03% of M_s_ for the bead after copper adsorption, compared to that before copper adsorption, is expected, but it is too low to explain the difference between the data in Figure 6 and Figure 7. After these evaluations, the difference in signal intensity obtained by comparing M(H) for the bead before and after copper adsorption cannot be explained by considering only the presence of copper ions or an underestimation of the mass of the magnetic component.

Copper adsorption also has another effect, which can be observed in the behavior of magnetization at high fields at 300 K. In Figure 7, the magnetization associated with the BNP1 sample before adsorption shows a maximum around 58,000 Oe and then decreases with the increase in the field. This behavior is also observed for the sample of BNP1 after copper adsorption (see Figure 7). However, the maximum has reached around 37,000 Oe. After this field value, magnetization starts to decrease until the maximum field is reached. This can be explained by considering the diamagnetic behavior of copper, which is relevant when the signal of magnetic nanoparticles reduces its growth. In fact, this behavior is observed for the M(H) at 300 K, but not for the M(H) at 5 K, in which the magnetization does not reach a maximum in either case, before and after copper adsorption.

**Figure 8:**
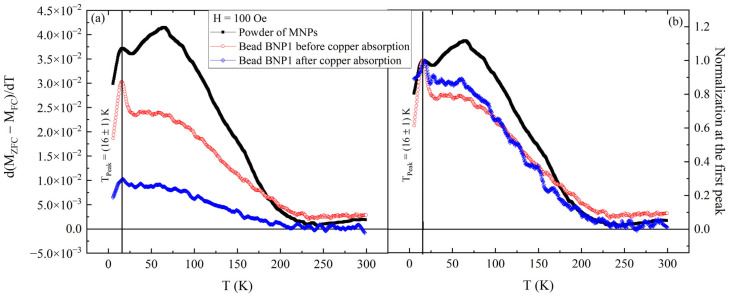
(**a**) Blocking temperature distribution extracted from the M(T) curves. The figure shows the comparison between the distributions obtained for the powder of Fe_3_O_4_ magnetic nanonoparticles (black closed squares), the dried BNP1 bead (red open circles), and the dried BNP1 bead after copper adsorption (blue crossed diamonds). Each distribution shows a peak at 16 K. On the right (**b**), the same curves are compared after normalization with the intensity of the peak at 16 K.

To investigate the signal reduction between Figure 6 and Figure 7, the distribution of blocking temperature extracted from the M(T) curves, as the temperature derivative of the difference between ZFC and FC curves, was studied for all three samples [27,30]. In Figure 8, the comparison among the three blocking distributions is shown. As can be seen in Figure 8a, the presence of a peak at 16 K is evident for all three samples. This peak in the blocking temperature distribution has the physical meaning of the average blocking temperature for the samples [24,27,28,30,31]. The presence of this peak for all the samples underlines the fact that the microscopic properties of magnetic nanoparticles remained the same and they were not affected by either the incorporation in the bead or the presence of copper. This is more evident when considering Figure 8b, in which each distribution is normalized with the intensity of the peak at 16 K. As can be seen, the shapes of the three distributions were very similar, except for the intensities of the peaks. In fact, the distribution extracted for the powder of magnetic nanoparticles showed two peaks, the first one at 16 K, as previously mentioned, and the second one at the temperature of 65 K. The presence of double peaks in the distribution of blocking temperature has different possible explanations in the literature, connected to either different interactions between nanoparticles present in the sample [24,32] or a different dimension distribution for them [33]. After the incorporation of the beads, the intensity of the peak at 65 K was reduced, but the peak at 16 K remained, as stated before. This determines a relative increase in the peak amplitude at 16 K compared to that at 65 K (Figure 8a). The reduction in the intensity of the peak at 16 K is in line with the loss of magnetic signal observed in the M(H) measurements in Figure 6. In fact, considering the mass magnetization from Figure 3, Figure 5 and Figure 6 at H = 100 Oe, the field used to perform the M(T)s, it is possible to observe a reduction of 27% in the signal between the powder of MNPs and BNP1 and a reduction of 75% in the signal between the powder of MNPs and BNP1 after copper adsorption. Comparing the signals in Figure 8a, it is possible to observe a similar trend for the peak at 16 K with a reduction of 20% between the powder of MNPs (black curve) and BNP1 before copper adsorption (red curve) and a reduction of 73% between the same samples after copper adsorption (blue curve). This trend is not the same for the peak at 65 K, since the signal reduction is 42% before copper adsorption and 80% after copper adsorption. Thus, the intensity variation of the peak at 65 K is more pronounced than that at 16 K for BNP1 after copper adsorption. This result also gives the information that the change in blocking temperature distribution is due to a reduction of the peak at 65 K. The point now is to understand what can determine a change in the distribution of magnetic nanoparticles’ properties in the bead. Following the results of Germanos et al. [26], copper adsorption in a bead of calcium alginate can change the spatial distribution of incorporated magnetic nanoparticles. Through a study of the beads via EDS, the authors observed that magnetic nanoparticles were perfectly distributed in the bead before copper adsorption, but after the adsorption, magnetic nanoparticles started to move from the center of the bead to its side regions. This not only changed the spatial concentration of magnetic nanoparticles, but it could also explain the change in the blocking distribution due to possible changes in MNPs interaction and aggregation. Considering these observations, the changes observed in Figure 8b after copper adsorption are in line with a variation of the MNPs configuration in the bead.

## 4. Conclusions

The magnetic measurements performed on a powder of Fe_3_O_4_ nanoparticles confirmed its superparamagnetic behavior at room temperature. This can be observed from either the change in coercivity measured at both 5 K and 300 K or the analysis of the M(T) measurement. The presence of a superparamagnetic state also remained when the nanoparticles were used in the synthesis process of a cross-linked sodium alginate bead. In particular, the saturation magnetization extracted from the M(H) measurement was lower than that measured for the pure MNPs powder, although of the same order of magnitude, confirming the possibility of using the bead in water remediation applications. The same characterization was performed on a bead dried after the adsorption of copper ions from a water solution. The variation in coercivity between the M(H) at 5 K and the M(H) at 300 K suggested that the superparamagnetic behavior still remained on the bead after copper adsorption. However, the saturation magnetization was somewhat reduced compared to that measured for the bead without copper adsorption and for the powder of magnetic nanoparticles. However, there is a significant reduction in the magnetic signal after the metal ion absorption. This reduction cannot be explained by considering the effect of the copper magnetic signal, and it can suggest a change in magnetic nanoparticle spatial configuration in the bead. This result was supported by the comparison of the blocking temperature distributions obtained from the M(T) measurements. The magnetization reduction is important from the perspective of applications because it can affect the efficiency of bead removal from the water medium. Starting with these results, the possibility of using magnetic signal measurements of the beads as a nondestructive determination of the adsorbent loading during use can be studied further.

## Figures and Tables

**Table 1:** Wet and dried mass of the different beads.

	BNP1		BNP2		BGONP		BA	
State	Wet	Dry	Wet	Dry	Wet	Dry	Wet	Dry
N. of Beads	27	27	29	29	34	34	20	20
Average Weight	23.4 mg	0.7 mg	23.1 mg	0.7 mg	22.6 mg	0.6 mg	15.9 mg	0.91 mg
Std. Dev.	2.5 mg	0.1 mg	1.6 mg	0.1 mg	3.6 mg	0.1 mg	0.91 mg	0.060 mg
Std. Dev. (%)	10.5%	12.4%	6.80%	13.1%	16.0%	13.4%	5.74%	12.0%

**Table 2:** Maximum load determined according to the Langmuir model for each case studied (R^2^ for each isotherm is reported in brackets).

pH = 3			pH = Unbuffered	
	25 °C	35 °C	45 °C	25 °C
Sample	Langmuir max load Q_max_ (mg/g_bead_)			
BNP1	264 (R^2^ = 0.8949)	197 (R^2^ = 0.9105)	164 (R^2^ = 0.7589)	220 (R^2^ = 0.9620)
BNP2	262 (R^2^ = 0.7855)	156 (R^2^ = 0.9062)	152 (R^2^ = 0.8982)	276 (R^2^ = 0.8549)
BGONP	218 (R^2^ = 0.7667)	155 (R^2^ = 0.8041)	141 (R^2^ = 0.7589)	354 (R^2^ = 0.9015)
BA	248 (R^2^ = 0.8754)	159 (R^2^ = 0.8553)	170 (R^2^ = 0.8735)	338 (R^2^ = 0.9206) 348 (R^2^ = 0.9262)

**Table 3:** Starting concentration and copper load on each sample of beads.

	Starting Cu^2+^ Concentration [g/L]			
	3.00	2.00	1.00	0.25
Sample	X_load_ of Cu^2+^ [mg/g_beads_]			
BNP1	180	134	115	20.2
BNP2	197	155	105	17.3
BGONP	300	240	147	86.4
BA	295	227	170	90.1

## Data Availability

Data are contained within the article.
